# Influence of Flexible Classroom Seating on the Wellbeing and Mental Health of Upper Elementary School Students: A Gender Analysis

**DOI:** 10.3389/fpsyg.2022.821227

**Published:** 2022-05-27

**Authors:** Jonathan Bluteau, Solène Aubenas, France Dufour

**Affiliations:** Département d’Éducation et Formation Spécialisées, Université du Québec à Montréal, Montréal, QC, Canada

**Keywords:** physical environment, classroom layout, flexible seating, wellbeing, mental health, upper elementary school

## Abstract

While traditional seating (also known as *fixed seating* or *fixed classroom*) remains the preferred classroom seating arrangement for teachers, a new type of seating arrangement is becoming more common in schools: the flexible classroom (also known as *flexible seating*). The purpose of this type of arrangement is to meet the needs of students by providing a wide variety of furniture and workspaces, to put students at the center of learning, and to allow them to make choices based on their preferences and the objectives of the task at hand. This study aimed to examine the influence of flexible seating on the wellbeing and mental health of elementary school students. This article presents the results of exploratory research conducted in Quebec among Grade 5 and 6 students comparing the wellbeing and mental health of students in fixed and flexible classrooms. The study was conducted with 107 students in three Grade 5 and 6 flexible classrooms (*n* = 51) and three Grade 5 and 6 fixed classrooms (*n* = 56). It is based on a quasi-experimental, quantitative design with post-test only and a control group. The groups were matched based on natural conditions (i.e., from a convenience sample). Furthermore, the study included a gender-differentiated analysis for each group. The results showed that flexible classroom seating had a positive influence on the girls’ wellbeing and mental health. In contrast, for the boys, fixed classroom seating was most conducive to their wellbeing and mental health. However, our study has some limitations that are discussed in the article.

## Introduction

Over the past few years, there has been a growing awareness in the education community about the importance of the school physical environment (Gouvernement du Québec. Ministère de l’Éducation et de l’Enseignement Supérieur, 2020). Indeed, by the possibility that students have to interact with the physical school environment through movement, exploration, and social interaction, it would strengthen the physical, cognitive, emotional, and social development of students based on the principle that a well thought-out school physical environment promotes the global development of students, promotes academic wellbeing and inclusion (Aziz et al., 2017). In fact, the fields of architecture and educational psychology have looked at the dimensions of the physical environment that can impact on the global development of students. According to an interdisciplinary perspective, the school space is thought of as a living environment where the student and his environment interact in a transactional way and mutually define each other (Jodelet, 2015). In this perspective, the theoretical model proposed by Pianta et al. (2008), on transactional and developmental theories, presents an interactional model between students, the teacher, and the school environment. The school environment is considered in all aspects of the daily experience and interactions that the student has with his teacher and his peers. As a result, the educational environment influences the resulting social interactions and the cognitive and socio-affective development of students (Bronfenbrenner and Morris, 1998; Broto, 2013; Huynh et al., 2013).

Numerous international publications (Organisation de coopération et de développement économiques, 2001, 2011) on the 21st-century school were pivotal in considering the role of the physical environment in students’ school experiences. As a result, many education systems, such as those in Quebec, France, Germany, Denmark, and Finland, have begun to ask whether the school environment in which students develop can contribute to their sense of wellbeing and, ultimately, to their success. At the same time, teachers are increasingly interested in the question of how to structure their classrooms to meet teaching requirements and support learning. The term “classroom physical environment” refers to all the furniture and its spatial arrangement in the classroom (Abbasi, 2013). We note that the majority of classroom arrangements, particularly in Quebec, remain fixed classrooms. However, in recent years, a new type of classroom seating arrangement has developed: the flexible classroom seating (Laquerre, 2018; Vallée, 2019).

The fixed classroom, also known as the traditional classroom, is the most commonly observed seating arrangement in schools and is also associated with teacher-centered practice. In this type of classroom, there are as many desks as there are students, and the teacher is usually responsible for assigning a desk to each student. Desk arrangement can vary—rows, U-shaped, or clusters. There are several explanations for the choice of desk arrangement. Desks arranged in rows especially encourage individual work (Wannarka and Ruhl, 2008), while U-shaped or cluster arrangements encourage social interaction and cooperation (Wannarka and Ruhl, 2008; Farmer et al., 2011; Gest and Rodkin, 2011). However, desk arrangement is rarely changed during the year, and classrooms are not routinely rearranged for a particular teaching activity.

The second type of arrangement, called the flexible classroom seating, is currently gaining traction with teachers (Laquerre, 2018; Vallée, 2019). In Quebec, it is estimated that there are more than 1,500 flexible classrooms in place throughout its School Services Centres (CSSs; Bluteau et al., 2019). Adoption of the flexible classroom has spread as a result of social networks (Havig, 2017). In this type of classroom, some or all of the desks have been replaced by a wide range of so-called flexible furniture that offers a variety work surfaces, seating sizes and heights, body positions (Dornfeld, 2016; Havig, 2017; Limpert, 2017; Del’Homme, 2018; Laquerre, 2018; Legout, 2018; Tiennot, 2019; Vallée, 2019). In this way, students do not have assigned seating (Legout, 2018). They can move about in the classroom and choose the seat that best suits them for the task at hand (Dornfeld, 2016; Havig, 2017; Limpert, 2017; Del’Homme, 2018; Laquerre, 2018; Legout, 2018; Tiennot, 2019; Vallée, 2019). It allows students to explore, move about, experiment, manipulate, and make the space and furniture their own, with the goal of encouraging original and creative ways of experiencing the classroom (Abbasi, 2013; Mazalto and Paltrinieri, 2013; Keymeulen et al., 2020). Flexible furniture is also designed so that classrooms can be modified easily. Teachers can therefore rearrange their classroom to suit the teaching activity and the type of behavior expected (Wannarka and Ruhl, 2008; Havig, 2017; Carignan, 2018; Erz, 2018; Keymeulen et al., 2020), including group work, pair work, or individual work. From this perspective, this type of classroom arrangement allows for implementing teaching practices that can be described as “flexible,” that is, student-centered, differentiated, and collaborative (Barrett et al., 2015, 2017; Delzer, 2015; Dornfeld, 2016; Havig, 2017; Erz, 2018; Keymeulen et al., 2020). Flexible classrooms address the principles of the “Current pedagogical discourse […] focused on learning, on putting the student at the center of the discussion, on helping them to be adaptive, creative, cooperative, responsive, and self-reliant.”^1^ (Blyth, 2013, p. 53). Thus, the flexible classroom seating is more associated with student-centered teaching practice.

Previous research has found that the functioning of the flexible classroom seating contributes to the development of certain personal skills, such as self-reliance, self-regulation, and problem-solving (Doyon, 2018; Erz, 2018; Laquerre, 2018; Legout, 2018). The flexible seating can therefore help to empower students and make them actors in their own learning (Legout, 2018). Furthermore, the functioning of the flexible classroom seating can have positive effects particularly on attention, motivation, engagement, and the adoption of task-appropriate behavior (Delzer, 2015; Dornfeld, 2016; Boudreault, 2017; Comaianni, 2017; Limpert, 2017; Allen, 2018; Erz, 2018; Laquerre, 2018; Legout, 2018; Schrage, 2018; Tiennot, 2019). By encouraging movement, choice, and interaction, and increasing students’ sense of control, this type of classroom arrangement addresses students’ physical, social, and cognitive needs (Comaianni, 2017; Havig, 2017; Limpert, 2017; Erz, 2018; Legout, 2018; Schoolcraft, 2018; Schrage, 2018; Sorrell, 2019; Vallée, 2019). However, there are limitations to flexible seating addressed in the literature that should be mentioned. On the one hand, this type of classroom arrangement may be challenging for students who need guidance and routine (Legout, 2018; Vallée, 2019). On the other hand, some students may be challenged by the lack of personal space. As Legout (2018) found, shared furniture and space, and no longer having an assigned desk, do not work for all students. Nevertheless, despite the growing interest by teachers in this type of arrangement, the flexible classroom remains poorly documented in the literature (Havig, 2017; Laquerre, 2018; Vallée, 2019), and its influence on student learning and mental health is still poorly understood. As a result, there is a sizeable gap between research that is currently available and the enthusiasm that this type of arrangement has generated among teachers (Havig, 2017; Laquerre, 2018; Vallée, 2019).

Furthermore, interest in school wellness and mental health came late to the field of studies in education (Piché et al., 2017) and has become prominent in many educational system reforms (Bacro et al., 2017). Indeed, the redefinition of success to include various aspects of students’ holistic development has made wellbeing a fundamental concept of the 21st-century school (Guimard et al., 2015; Ferrière et al., 2016). Numerous studies have revealed that lived school experiences are associated with development, identity construction, academic success, and wellbeing (Konu and Rimpelä, 2002; Wigfield et al., 2006; Eccles and Roeser, 2011; Rousseau, 2012; Bacro et al., 2017). In this sense, wellbeing at school may depend on many factors rooted in students’ school experience (Guimard et al., 2015, as cited in Fouquet-Chauprade, 2013; Ferrière et al., 2016). Wellbeing is a multidimensional, multifactorial, and systemic concept (Conseil Supérieur de l’Éducation, 2020). It has been characterized according to objective, subjective, environmental, and contextual factors (Espinosa and Rousseau, 2018). The scientific literature takes two divergent paths to define wellbeing. On the one hand, the hedonic conception associates wellbeing with pleasure, satisfaction, and subjective happiness (Laguardia and Ryan, 2000; Doré and Caron, 2017; Conseil Supérieur de l’Éducation, 2020). Thus, a positive sense of wellbeing “consists of experiencing many positive affects, few unpleasant ones” (Laguardia and Ryan, 2000, p. 282), but also “feeling a high overall satisfaction with one’s life”^2^ (Florin and Guimard, 2017, p. 20). On the other hand, the eudemonic conception of wellbeing refers to personal fulfillment and self-actualization (Laguardia and Ryan, 2000; Conseil Supérieur de l’Éducation, 2020). Commonly referred to as psychological wellbeing, this conception is more recent (Antoine et al., 2007). Here, wellbeing consists solely in living in accordance with one’s own nature and values (Laguardia and Ryan, 2000; Conseil Supérieur de l’Éducation, 2020). For a long time, these two approaches have represented divergent directions for research. However, at present, a combination of the two conceptions would seem necessary to encompass wellbeing in its entirety: “Well-being should be understood as a state of subjective pleasure and satisfaction with life, but also of self-actualization”^3^ (Conseil Supérieur de l’Éducation, 2020, p. 20). This more encompassing definition comes close to the current World Health Organization (WHO) definition of good mental health. Indeed, the rise of positive psychology has led to a more encompassing definition of mental health (Ferrière et al., 2016; Doré and Caron, 2017; Shankland et al., 2017). This new branch in psychology is defined as, “the scientific study of positive experiences, wellbeing, and optimal functioning of the individual”^4^ (Antoine et al., 2007, p. 170). The definition of positive mental health, also known as optimal mental health, takes into account the wellbeing and good psychological and social functioning of the individual (Doré and Caron, 2017; Shankland et al., 2017; Conseil Supérieur de l’Éducation, 2020). Indeed, mental health includes all dimensions of a student’s overall development (Welsh et al., 2015) and can be defined by low stress, a sense of psychological wellbeing, and ultimately, good coping and behavioral functioning. Thus, mental health and wellbeing are closely linked (Conseil Supérieur de l’Éducation, 2020). Protective factors that positively influence mental health and wellbeing in school and decrease exposure to stressors include the quality of the physical environment, classroom interactions (Amoly et al., 2014), and social support provided by the teacher (Kruger et al., 2007; Heaney and Israel, 2008). However, the wellbeing, and ultimately, the mental health of students can be influenced by the quality of the environments they occupy, which can be explained by “the degree to which psychological and/or physiological needs are met in each environment, the physical perception of the environment, and the atmosphere of the environment” (Joing et al., 2018, p. 19). Furthermore, few studies to our knowledge have examined the relationship between classroom seating arrangement and student wellbeing and mental health. To date, the few studies that have examined the effects of seating arrangement show that certain aspects of the school physical environment (natural light, space, air quality, and so on) are linked to student concentration in the classroom and academic achievement (Cheryan et al., 2014; Barrett et al., 2017). However, in studies related to school architecture, little attention has been given to student mental health and wellbeing in the classroom. Nevertheless, seating arrangement and furniture play a role in teaching situations and overall development, and may ultimately influence student wellbeing and mental health (Mazalto, 2017; Doyon, 2018; Erz, 2018; Joing et al., 2018; Laquerre, 2018; Legout, 2018). As such, examining the influence of seating arrangement on mental health and wellbeing by investigating two types of classroom arrangements (fixed classroom seating and flexible classroom seating) may fill a gap in the existing literature on the topic, which this study has sought to do.

With this in mind, the question that guided this study was “Does classroom seating influence the academic wellbeing and mental health of elementary school students?” To answer this question, the study had two specific objectives: (1) compare the wellbeing and mental health of Grade 5 and 6 students in the context of differentiated classroom arrangements (fixed and flexible classrooms); and (2) compare, by gender, the wellbeing and mental health of Grade 5 and 6 students in the context of differentiated classroom arrangements (fixed and flexible classrooms).

Our starting hypothesis was to observe differences between the groups on the variables studied and an increase of wellbeing and better mental health in the flexible group, with no difference in terms of gender.

## Materials and Methods

### Research Design

This study is part of the Social Sciences and Humanities Research Council-funded research project “Influence of Classroom Seating Arrangement and Quality of Teacher-Student Interactions on Stress Coping and School Mental Health of Elementary School Students” (Bluteau et al., 2019). The study was approved by the Institutional Human Research Ethics Committee (CIEREH) of the Université du Québec à Montréal (UQAM) in October 2019. The study bears the following ethics certificate number: 3761_e_2019. The CIEREH agreement was then sent to the head office of the School Services Centre (CSS) for verification and approval.

The study is based on a quasi-experimental, quantitative design with post-test only and a control group. The groups were matched based on natural conditions (i.e., from a convenience sample). Participants were Grade 5 and 6 students in three fixed and flexible classrooms. Wellbeing and mental health were studied in both groups, and for boys and girls in each group.

### Participants

#### Teachers

Although teachers were not the focus of the study, teachers are in charge of the classroom and subject to its design. Thus, for research validity, it was important to select teachers in such a way as to control for teacher effect. Teacher effect can be perceived through the teacher’s attitude, experience, and sense of efficacy, among others. For this reason, a questionnaire was given to each teacher who wanted to participate. The purpose of the questionnaire, whose variables will be described in Section “Measuring Instruments and Data Collection Procedures,” was to match the three flexible classroom teachers to three fixed classroom teachers with similar teacher profiles in terms of sense of self-efficacy, job satisfaction, intention in the performance goal structure, years of teaching experience, and age. In the end, each flexible classroom teacher was matched to three fixed classroom teachers based on scale score equivalence. Consequently, six female teachers agreed to have students in their classrooms participate. The mean age of the female teachers in the fixed classrooms was 36 (SD = 6.36), and in the flexible classrooms (SD = 2.65) it was 34. As such, two fixed Grade 5 classrooms and one fixed Grade 6 classroom were matched to two flexible Grade 5 classrooms and one flexible Grade 6 classroom. In this way, each pair of teachers taught at the same level, had the same profile, and were in the same age range.

#### Students

The sample consisted of 107 students: 51 students in fixed classrooms (24 girls and 27 boys) and 56 students in flexible classrooms (26 girls and 30 boys). We note that the number of students in each group and the proportion of girls to boys in each group were relatively equal. The mean age of students in the fixed classrooms was 11.13 (SD = 0.54). Students in the flexible classrooms had a mean age of 11.23 (SD = 0.7; see Table 1).

**Table 1:** Sample description.

	Total	Fixed group	Flexible group
Student data		51	56
Age; Mean (SD)	107	11.13 (0.5)	11.23 (0.7)
Girls	50	24	26
Boys	57	27	30

#### Procedures

To limit bias, the study ensured that the two groups of students were equivalent on multiple levels, namely, (1) the School Services Centre, (2) the teachers, (3) the classes, and (4) the students.

The first phase consisted of establishing a partnership with a Montreal South Shore School Services Centre (CSS). The six classes in the sample were drawn from the same CSS. Notably, the CSS had been undertaking numerous expansion and construction projects to redesign classrooms and schools. Furthermore, the CSS was implementing flexible classrooms in a controlled manner to document the testing of this type of classroom and to examine its impact on indicators of educational success.

In the second phase, initial contact was made with the teachers of the partner CSS. Approximately 50 CSS teachers were contacted by email. The objective was to recruit teachers interested in the project who were teaching Grade 5 and 6 students in flexible classrooms. They were asked if they were interested in the project and, if so, whether they taught in a flexible classroom environment with Grade 5 and 6 students. As a result, three flexible classroom teachers who met the criteria were selected. Subsequently, a CSS manager in charge of the study was able to provide us with contact information for eight Grade 5 and 6 teachers (one male and seven female teachers) who had opted for the fixed classroom arrangement. A matching questionnaire (see “Teacher Matching”) was distributed to the three flexible classroom teachers and the eight fixed classroom teachers in order to pair the three flexible classroom teachers with three fixed classroom teachers based on a number of criteria. A total of seven fixed classroom teachers responded to the questionnaire, three of whom were matched to the three flexible classroom teachers.

Next, we examined the socio-economic background index for each school in our sample. The schools in our sample were public schools located in rural and semi-rural areas. The Disadvantaged Index for all public elementary and secondary schools, made available by the Ministère de l’Éducation et de l’enseignement supérieur (Ministry of Education and Post-Secondary Studies), provided a picture of the socio-economic background index (IMSE) for each school in our sample. Schools are ranked on a scale from 1 to 10, with a score of 1 representing the least disadvantaged schools and a score of 10 representing the most disadvantaged schools (Gouvernement du Québec. Ministère de l’Éducation et de l’Enseignement Supérieur, 2020; see Table 2).

**Table 2:** Socio-economic environment index (IMSE) and decile rank for each school in the sample.

Classroom	Grade	School	Socio-economic environment index (IMSE)	Decile rank (IMSE)
Fixed group				
Classroom 1	5	School 1	9.52	7
Classroom 2	5	School 1	9.52	7
Classroom 3	6	School 2	9.15	6
Flexible group				
Classroom 4	5	School 3	3.79	2
Classroom 5	5	School 4	5.50	3
Classroom 6	6	School 5	8.77	6

Two fixed classrooms were in the same school having a score of 7. The school of the remaining fixed classroom had a score of 6. Consequently, these classrooms were in the least advantaged schools in the CSS. As for the flexible classroom schools, one had the same disadvantage index score as the fixed classroom schools (6). However, the other two schools with flexible classrooms had scores of 2 and 3, respectively, meaning they were in the most advantaged schools in the CSS. Therefore, the schools were not equivalent overall with regard to their disadvantage index.

After receiving approval from the Institutional Human Research Ethics Committee (CIEREH) and authorization from the CSS, the school principals, and the teachers, we were able to meet with the students in class to present the study. Since participation in the project was voluntary, an invitation to participate and a consent form were distributed for parents to sign. Data collection took place during December 2019 (pre-pandemic). As such, the students were exposed to the research environment for a period of 4 months, from the beginning of the school year. The data collection procedure lasted, on average, about 40 min per student and was conducted under the supervision of a research assistant. Students were asked to complete two questionnaires (see “Students”): the Liddle and Carter (2015) questionnaire and the BASC-3 (Reynolds and Kamphaus, 2015). The Liddle and Carter (2015) questionnaire was distributed in paper format and took students 10 min on average to complete. The BASC-3 required 30 min on average to complete. Students completed the paper version, and their responses were transcribed using the Q-Global platform licensed by NCS Pearson, Inc. No participants withdrew during data collection.

### Measuring Instruments and Data Collection Procedures

#### Teacher Matching

As mentioned above, the objective of the questionnaire was to match the three flexible classroom teachers with three fixed classroom teachers having a similar teacher profile in order to control for teacher effect. The self-report questionnaire consisted of twenty-four items divided into three categories. The *Teacher Self-Efficacy scale* consisted of ten items (*α* = 0.82; Schwarzer, 1992; Bandura, 1997; Schwarzer and Hallum, 2008) in which teachers were asked to choose among four Likert scale responses (not at all true, only slightly true, moderately true, completely true). *Job accomplishment* was assessed using five items (*α* = 0.77; Ho and Au, 2006). Teachers were asked to select the most appropriate response among five items (strongly disagree, somewhat disagree, neither disagree nor agree, somewhat agree, strongly agree). Finally, *Intention in the performance goal structure* was measured using nine items (*α* = 0.69; Midgley et al., 2000). Teachers were asked to rate each statement on a Likert scale from 1 to 7, 1 being completely false and 7 being completely true. The questionnaire took an average of 10 min to complete.

#### Students

Students were asked to complete two self-reported questionnaires individually, the first measuring wellbeing at school using the Liddle and Carter (2015) questionnaire, and the second measuring mental health using the BASC-3 tool (Reynolds and Kamphaus, 2015), which reports on students’ coping and behavioral functioning.

The Liddle and Carter (2015) questionnaire is a 12-item self-report questionnaire (*α* = 0.82) using a Likert scale (never, not often, regularly, often, all the time). For example, students were asked to respond to the following statements: “I think good things will happen to me in my life”; “I get along with people”; and “I feel relaxed.”^5^

The BASC-3 (Reynolds and Kamphaus, 2015) measurement tool examines the mental health of students by taking into account various aspects of personal adjustment and behavioral functioning. It consists of 137 items. The five composite scales (*internalizing problems, inattention/hyperactivity, school problems, emotional symptoms, personal adjustment*; *α* = 0.89–0.95) grouped ten clinical scales (*anxiety, attention problems, attitude to school, attitude to teachers, atypicality, depression, hyperactivity, locus of control, sense of inadequacy, social stress*; *α* = 0.73–0.86) and four adaptive scales (*relations with parents, interpersonal relations, self-esteem*, *self-reliance*; *α* = 0.75–0.87; see Table 3). The questionnaire was divided into two parts. In the first part, students were to answer true or false for each statement. For example, students were asked to respond to the following statements: “I often do things without thinking”; “I am not interested in school”; “I like who I am.”^6^ In the second part, students were to choose from four Likert scale items (never, sometimes, often, almost always). In this section, students were asked, among other things, to respond to the following statements: “I get along well with others”; “I am nervous”; and “I am a good listener.”^7^

**Table 3:** Description of variables measured by BASC-3 (Reynolds and Kamphaus, 2015).

Internalizing problems	Inattention/ hyperactivity	School problems	Emotional symptoms	Personal adjustment
Atypicality; Locus of control; Social stress; Anxiety and depression; Sense of inadequacy	Attention problems; Hyperactivity	Attitude to school; Attitude to teachers	Social stress; Anxiety and Depression; Sense of inadequacy; Self-esteem; Self-reliance	Relations with parents; Interpersonal relations; Self-esteem; Self-reliance

### Data Analysis

To provide a portrait of each group, descriptive analyses were performed on the entire sample (*N* = 107) using the two variables of “age” and “gender.” For each group, age was described by mean and standard deviation (SD). The “gender” variable was described by its frequency in each group (fixed and flexible classrooms).

As previously mentioned, the students’ responses to the BASC-3 were transcribed using the Q-Global platform, thus providing an analysis of all students’ scores. Scores were standardized, that is, raw scores were translated into T-scores and percentile scores (see Table 4).

**Table 4:** Basc-3 scale and composite score classification (Reynolds and Kamphaus, 2015).

T-score Range	Clinical Scales	Adaptive Scales
70 and above	Clinically significant	Very high
60–69	At risk	High
41–59	Average	Average
31–40	Low	At risk
30 and below	Very low	Clinically significant

Normality of distribution was verified beforehand using a normal probability plot with Henry’s line, and homogeneity of variance was verified by a Levene test. For statistical analyses, STATA 15.1 software was used, with a significance level less than or equal to 0.05. A Student *t*-test was performed (independent variable “group” with two categories) to analyze the different variables measured by the instruments (quantitative dependent variables). Differential analyses were also conducted by gender (boys and girls separately). The results of the study are discussed in the next section.

## Results

To recall, the purpose of this exploratory study was to compare the wellbeing and mental health of Grade 5 and 6 students in differentiated classroom seating arrangements (fixed and flexible classrooms). Secondly, the study compared gender-specific wellbeing and mental health of Grade 5 and 6 students in differentiated classroom seating arrangements (fixed and flexible classrooms). The results are therefore presented in three parts:

1. Mental health indicator results by group (fixed and flexible) for both genders.
2. Mental health indicator scores by group (fixed and flexible) for boys.
3. Mental health indicator scores by group (fixed and flexible) for girls.

### Mental Health Indicator Results by Group (Fixed and Flexible) for Both Genders

Table 5 presents the mental health indicator scores by group (fixed and flexible) for both genders. For each mental health indicator, the mean (M), standard deviation (SD), minimum, and maximum obtained by students in each group, as well as statistical significance (*p*-value), are presented.

**Table 5:** Mental health indicator results by group (fixed and flexible) for both genders.

	Indicator	Mean (SD)	Minimum–Maximum	*p*-value
Fixed group	Wellbeing (*n = 51*)	54.8 (9.57)	33.5–72.0	0.197
	Mental health (*n = 51*)			
	Internalizing problems (*n = 51*)	53.1 (11.7)	35.0–79.0	0.740
	Inattention/hyperactivity (*n = 51*)	50.9 (9.30)	35.0–73.0	0.618
	School problems (*n = 51*)	49.3 (9.19)	37.0–74.0	0.426
	Emotional symptoms (*n = 51*)	51.5 (11.0)	35.0–77.0	0.901
	Personal adjustment (*n = 51*)	52.3 (7.66)	29.0–63.0	0.963
Flexible group	Wellbeing (*n = 56*)	57.1 (8.57)	39.0–74.0	0.197
	Mental health (*n = 56*)			
	Internalizing problems (*n = 56*)	52.4 (10.5)	36.0–90.0	0.740
	Inattention/hyperactivity (*n = 56*)	51.9 (11.0)	34.0–75.0	0.618
	School problems (*n = 56*)	48.0 (8.04)	37.0–74.0	0.426
	Emotional symptoms (*n = 56*)	51.3 (10.6)	36.0–92.0	0.901
	Personal adjustment (*n = 56*)	51.5 (9.46)	23.0–63.0	0.963

For the “wellbeing” variable, the difference was not statistically significant (*p* = 0.197). Students in the flexible group had a lower mean wellbeing score of 57.1 points (SD = 8.57) compared to a mean wellbeing score of 54.8 points (SD = 9.57) for students in the fixed group.

For the “internalizing problems” variable, students in the fixed group had a slightly higher mean score (*M* = 53.1; SD = 11.7) than students in the flexible group (*M* = 52.4; SD = 10.5). Although this difference was not statistically significant (*p* = 0.740), the scores suggest that students in the flexible group exhibited less atypicality, social stress, anxiety, and depression. They appeared to have a better locus of control and a lower sense of inadequacy compared to students in the fixed group.

For the “inattention/hyperactivity” variable, students in the fixed group had a mean score of 50.9 points (SD = 9.30), while students in the flexible group had a mean score of 51.9 points (SD = 11.0). Thus, students in the flexible group reported relatively more attention problems with or without hyperactivity than students in the fixed group. However, this difference was not statistically significant (*p* = 0.618).

As for the “school problems” variable, which reflects student attitudes to school and teachers, the mean scores were 49.3 points (SD = 9.19) for students in the fixed group and 48.0 points (SD = 8.04) for students in the flexible group. However, this difference was not statistically significant (*p* = 0.426).

For the “emotional symptoms” variable, there were no statistically significant differences (*p* = 0.901) between the two groups. The mean scores were 51.5 points (SD = 11.0) for students in the fixed group and 51.3 points (SD = 10.6) for students in the flexible group. The students therefore had comparable mean scores for social stress, anxiety, depression, and sense of inadequacy, as well as self-esteem and self-reliance.

The difference was not statistically significant (*p* = 0.963) for the “personal adjustment” variable. Students in the fixed group had a score of 52.3 points (SD = 7.66), while students in the flexible group had a mean score of 51.5 points (SD = 9.46). This suggests that students in the flexible group had relatively better parent–child and interpersonal relations, higher self-esteem, and higher levels of self-reliance.

### Mental Health Indicator Results by Group (Fixed and Flexible) for Boys

Table 6 presents the mean scores [standard deviation (SD), minimum, and maximum] for boys’ mental health indicators by group (fixed and flexible), as well as the statistical significance (*p*-value) of each indicator.

**Table 6:** Mental health indicator results by group (fixed and flexible) for boys.

	Indicator	Mean (SD)	Minimum–Maximum	*p*-value
Fixed group	Wellbeing (*n = 27*)	55.3 (9.84)	33.5–72.0	0.492
	Mental health (*n = 27*)			
	Internalizing problems (*n = 27*)	50.2 (9.86)^*^	35.0–79.0	0.016
	Inattention/hyperactivity (*n = 27*)	50.7 (7.99)^**^	36.0–67.0	0.010
	School problems (*n = 27*)	53.1 (9.25)	39.0–74.0	0.454
	Emotional symptoms (*n = 27*)	48.9 (8.69)^*^	35.0–71.0	0.015
	Personal adjustment (*n = 27*)	52.9 (6.30)	41.0–63.0	0.064
Flexible group	Wellbeing (*n = 30*)	53.6 (9.02)	39.0–74.0	0.492
	Mental health (*n = 30*)			
	Internalizing problems (*n = 30*)	56.8 (10.3)^*^	38.0–90.0	0.016
	Inattention/hyperactivity (*n = 30*)	57.2 (10.3)^**^	37.0–75.0	0.010
	School problems (*n = 30*)	51.3 (8.51)	39.0–74.0	0.454
	Emotional symptoms (*n = 30*)	55.6 (11.1)^*^	38.0–92.0	0.015
	Personal adjustment (*n = 30*)	47.2 (10.4)	23.0–63.0	0.064

* *p ≤ 0.050;*

** *p ≤ 0.010*.

Regarding the “wellbeing” variable, although the difference was not statistically significant (*p* = 0.492), boys in the fixed group had a higher mean score (*M* = 55.3; SD = 9.84) than those in the flexible group (*M* = 53.6; SD = 9.02).

The difference was statistically significant (*p* = 0.016) for the “internalizing problems” variable. In the flexible group, boys had a higher mean score (*M* = 56.8; SD =10.3) than boys in the fixed group (*M* = 50.2; SD = 9.86). As a result, boys in the fixed group reported less atypicality, social stress, anxiety, and depression, and had a better locus of control and a lower sense of inadequacy compared to boys in the flexible group.

The mean scores for the “inattention/hyperactivity” variable were 50.7 points (SD = 7.99) for boys in the fixed group and 57.2 points (SD = 10.3) for boys in the flexible group. Therefore, boys in the fixed group presented less attention problems with or without hyperactivity than boys in the flexible group. The difference was statistically significant (*p* = 0.010).

As for the “school problems” variable, boys in the flexible group (*M* = 51.3; SD = 8.51) had relatively better attitudes to school and teachers compared to boys in the fixed group (*M* = 53.1; SD = 9.25). However, this difference was not statistically significant (*p* = 0.454).

For the “emotional symptoms” variable, the difference between the two groups was statistically significant (*p* = 0.015). The mean scores were 48.9 points (SD = 8.69) for boys in the fixed group and 55.6 points (SD = 11.1) for boys in the flexible group. Thus, boys in the flexible group had more social stress, anxiety, and depression. They also had a greater sense of inadequacy, lower self-esteem, and lower levels of self-reliance than students in the fixed group.

Regarding the “personal adjustment” variable, although the difference was not statistically significant (*p* = 0.064), boys in the fixed group had a higher mean score (M = 52.9; SD = 6.30) than boys in the flexible group (*M* = 47.2; SD = 10.4). Boys in the fixed group tended to have better interpersonal and family relationships, self-esteem, and self-reliance compared to boys in the flexible group, although the observed difference did not reach statistical significance.

### Mental Health Indicator Results by Group (Fixed and Flexible) for Girls

Table 7 presents the scores (mean, standard deviation [SD], minimum, and maximum) for mental health indicators by group (fixed and flexible) for girls, as well as the statistical significance (*p*-value) of each indicator.

**Table 7:** Mental health indicator results by group (fixed and flexible) for girls.

	Indicator	Mean (SD)	Minimum–Maximum	*p*-value
Fixed group	Wellbeing (*n = 24*)	54.2 (9.44)^**^	39.0–72.0	0.003
	Mental health (*n = 24*)			
	Internalizing problems (*n = 24*)	56.3 (12.9)^**^	38.0–75.0	0.004
	Inattention/hyperactivity (*n = 24*)	51.2 (10.8)^*^	35.0–73.0	0.050
	School problems (*n = 24*)	45.1 (7.19)	37.0–60.0	0.607
	Emotional symptoms (*n = 24*)	54.5 (12.7)^**^	38.0–77.0	0.007
	Personal adjustment (*n = 24*)	51.7 (9.04)	29.0–62.0	0.073
Flexible group	Wellbeing (*n = 26*)	61.1 (5.96)^**^	50.0–70.0	0.003
	Mental health (*n = 26*)			
	Internalizing problems (*n = 26*)	47.0 (8.01)^**^	36.0–68.0	0.004
	Inattention/hyperactivity (*n = 26*)	45.8 (8.33)^*^	34.0–68.0	0.050
	School problems (*n = 26*)	44.1 (5.46)	37.0–54.0	0.607
	Emotional symptoms (*n = 26*)	46.3 (7.58)^**^	36.0–77.0	0.007
	Personal adjustment (*n = 26*)	56.3 (4.87)	45.0–63.0	0.073

* *p ≤ 0.050;*

** *p ≤ 0.010*.

For the “wellbeing” variable, girls in the flexible group had a higher mean score (*M* = 61.1; SD = 5.96) than girls in the fixed group (*M* = 54.2; SD = 9.44). This difference between the two groups of girls was statistically significant (*p* = 0.003).

There was a statistically significant difference between the two groups of girls for the “internalizing problems” variable (*p* = 0.004). In the fixed classrooms, girls had a higher mean score (*M* = 56.3; SD = 12.9) than girls in the flexible classes (*M* = 47.0; SD = 8.01). Based on the results, girls in the fixed classrooms showed more internalizing problems than girls in the flexible classrooms. Thus, girls in the fixed group showed more atypicality, had greater social stress, anxiety, depression, and sense of inadequacy, and had poorer locus of control compared to girls in the flexible group.

For the “inattention/hyperactivity” variable, the difference was statistically significant (*p* = 0.050). Girls in the flexible group (*M* = 45.8; SD = 8.33) had fewer attention problems with or without hyperactivity than girls in the fixed group (*M* = 51.2; SD = 10.8).

Regarding school problems, girls in the fixed group were found to have a slightly higher mean score (*M* = 45.1; SD = 7.19) compared to girls in the flexible group (*M* = 44.1; SD = 5.46). Although the difference did not reach statistical significance (*p* = 0.607), the mean score for school problems for girls in the flexible group was lower (−1 point).

For the “emotional symptoms” variable, girls in the flexible group (*M* = 46.3; SD = 7.58) had a lower mean score compared to girls in the fixed group (*M* = 54.5; SD = 12.7) and thus had less social stress, anxiety, depression, had a lower sense of inadequacy, and had higher self-esteem and self-reliance than girls in the fixed group. This difference was statistically significant (*p* = 0.007).

The mean scores for the “personal adjustment” variable were 51.7 points (SD = 9.04) for girls in the fixed group and 56.3 points (SD = 4.87) for girls in the flexible group. Although this difference was not statistically significant (*p* = 0.073), girls in the fixed group tended to have poorer interpersonal and family relationships, self-esteem, and self-reliance.

## Discussion

The results presented in the previous section are discussed below. First, we will answer the research question. The results will then be discussed in light of the available scientific literature. Finally, the study’s contributions, limitations, and prospects for research will be discussed.

### Summary of Results and Answer to the Research Question

With regard to the above results, the comparative analysis of the two groups (fixed and flexible) for both genders combined did not reveal any statistically significant difference for the various variables. However, when the two groups were analyzed by gender, statistically significant differences were found between the two groups (fixed and flexible). The mean scores for boys showed statistically significant differences for the following variables: (1) internalizing problems, (2) inattention and hyperactivity, and (3) emotional symptoms. Thus, boys in the fixed group had significantly fewer internalizing problems, attention problems with or without hyperactivity, and emotional symptoms than boys in the flexible group. For girls, the statistically significant variables were (1) wellbeing, (2) internalizing problems, (3) inattention and hyperactivity, and (4) emotional symptoms. Unlike the boys, girls in the flexible group reported greater wellbeing and fewer internalizing problems, attention problems with or without hyperactivity, and emotional symptoms. Based on the results of our gender-differentiated analysis, it appears that classroom seating arrangement influenced the wellbeing and mental health of elementary students at school. Based on the data, boys had a greater sense of wellbeing and mental health in fixed classrooms. In contrast, among the girls, the classroom seating arrangement most conducive to their wellbeing and mental health, according to these results, was flexible seating. Thus, flexible seating seemed to be a real challenge for some students and a real asset for others, which we will now discuss.

### Flexible Seating: Advantages and Limitations

As noted above, previous research on flexible classroom seating has reported that this type of arrangement helps meet students’ needs (Comaianni, 2017; Havig, 2017; Limpert, 2017; Erz, 2018; Legout, 2018; Schoolcraft, 2018; Schrage, 2018; Sorrell, 2019; Vallée, 2019) and encourages the development of skills, such as self-reliance, self-regulation, and problem-solving (Doyon, 2018; Erz, 2018; Laquerre, 2018; Legout, 2018). Although flexible seating is intended to be student-centered and needs-based, our results indicate that this type of arrangement can be detrimental to the wellbeing and mental health of some students. In the flexible classroom, students no longer have a place assigned to them (Legout, 2018). They move about freely and choose the seat that best suits the task at hand. As a result, the flexible classroom requires students to apply more skills, such as self-control, problem-solving, self-reliance, cooperation, and soft skills, such as working together, and so on. Flexible seating may therefore require students to initially have good coping strategies.

Surveys in Quebec have reported that girls perform better in problem-solving and self-control skills, among other things (Direction régionale de santé publique de Montréal, 2018; Institut de la statistique du Québec, 2018). However, problem-solving is a critical coping skill in the flexible classroom since students are required to make strategic choices throughout the day (Dornfeld, 2016; Havig, 2017; Limpert, 2017; Del’Homme, 2018; Laquerre, 2018; Legout, 2018; Tiennot, 2019; Vallée, 2019). As for the skill of self-control, it allows students to self-regulate more readily (Félouzis, 1993; Bouchard et al., 2006; Besnard et al., 2016) and makes it easier to adapt to the norms and expectations of the school (Commissariat général à la stratégie et à la prospective, 2014; Esperbès-Pistre et al., 2015). Self-control is especially important in flexible seating to be able to exercise self-reliance and cooperation in a classroom where all the furniture is available to the students. In addition, it appears that girls tend to develop more pro-social behaviors conducive to cooperation in the classroom (Félouzis, 1993; Bouchard et al., 2006; Ruel, 2010; Besnard et al., 2016), a key aspect of the flexible classroom (Del’Homme, 2018). Thus, girls may have an easier time adapting and engaging in the flexible classroom, which would explain why girls in the flexible classrooms had a higher sense of wellbeing and mental health, as measured. More broadly, flexible seating, through the practices, behaviors, and attitudes it encourages, may benefit students who initially have good coping strategies, may enhance their sense of control and may help meet their needs (self-reliance, socialization, and so on). This type of classroom arrangement may therefore be conducive to their wellbeing and mental health.

However, students who have difficulty adapting and behaving in a way that is conducive to the task at hand may be challenged by the flexible classroom. Many studies have reported that boys are more affected by behavioral and learning problems (Walker and Berthelsen, 2007; Childs and McKay, 2010; Besnard et al., 2016), which may affect their concentration, on-task behavior (Félouzis, 1991, 1993; Bouchard et al., 2006; Walker and Berthelsen, 2007; Ruel, 2010; Girardin, 2012; Gilles, 2018), and coping skills. Some studies have noted that freedom of choice and movement can be challenging for students who need a framework and routine (Legout, 2018; Schoolcraft, 2018; Vallée, 2019). For these students, fixed seating appears to be beneficial to their wellbeing and mental health. This may be because having an assigned desk, in other words, a space of their own, is reassuring (Legout, 2018) and reinforces their sense of control. Moreover, fixed seating provides a framework that may be more appropriate for these students (Legout, 2018; Vallée, 2019). Thus, it is not so much flexible furniture *per se* that may explain why these students have a lower sense of wellbeing and mental health, but how the flexible classroom itself functions (less controlling environment, undefined personal space, and so on; Havig, 2017; Legout, 2018; Vallée, 2019). Nevertheless, flexible seating is not to be ruled out for students with coping difficulties, but it does require teachers to provide alternatives and more ongoing support for these students.

### Contributions of the Study

These results add to current knowledge in the field of educational research. Because flexible seating is a recent phenomenon, few studies have been conducted on the topic (Havig, 2017; Laquerre, 2018; Vallée, 2019) and little is known about the influence of flexible seating on student wellbeing and mental health. Furthermore, few studies have compared the two types of seating arrangements (fixed versus flexible classrooms), and those that do rarely conduct gender-differentiated analyses.

On a practical level, this study provides additional guidance for teachers. It invites teachers to better anticipate the potential limitations of flexible seating to better prepare students for change. Indeed, regular support by teachers for students who need to develop coping strategies would seem vital.

### Limitations of the Study

Some of the limitations of our study relate to our sample. First, the small sample size (*N* = 107) does not allow drawing generalizable conclusions from our results. Indeed, this study was exploratory and intended to generate hypotheses and research questions in a new field of research. Another limitation of our sample lies in the socio-economic background indices (IMSE). Although our study only considered the socio-economic factor of school in his neighborhood, it did not consider the socio-economic factor of each family. This study was part of an exploratory process at the start, and we did not plan to collect this data directly from the parents of students. Thus, concerning the distribution of the presence of psychological difficulties (e.g., internalized and externalized problems and ADHD) in the two groups, there could be misleading reading of the results.

Furthermore, recall that the schools in the fixed group were among the least advantaged schools in the CSS, while two schools in the flexible group were among the most advantaged schools. However, previous research indicates that difficult socio-economic conditions in the home environment are associated with lower sense of wellbeing, mental health, and academic achievement (Ayotte et al., 2009; Riberdy et al., 2013; Couture, 2019). According to Riberdy et al. (2013), youth from disadvantaged backgrounds are more likely to be diagnosed with a mental health problem and report a perceived mental health problem. As a result, a higher prevalence of behavioral problems (hyperactivity, internalizing, and externalizing problems) is observed in these youth (Ayotte et al., 2009; Riberdy et al., 2013; Kettani et al., 2017; Couture, 2019). Moreover, according to Childs and McKay (2010), boys appear to be more susceptible to the effects of a low socio-economic background. Thus, the poorer mental health of boys in the flexible group compared to boys in the fixed group does not appear to be explained by socio-economic background.

A number of limitations of the study relate to methodology. To better understand the results, it would have been useful to use a mixed design and incorporate qualitative data through individual or group interviews. Also, our starting methodology had to have two measurement times, which would have been optimal for answering our research questions. This constitutes a significant limitation to our study and to the interpretation of the results concerning the differences between our groups. Moreover, it would have been useful to do a second measurement at the end of the year (outside of the pandemic context) to compare the two groups over the school year and to see if there were any changes in mental health indicators. In this sense, an important limit of the results indicates that the independent variable (type of arrangement) has a significant effect, not so much on the processes of adaptation/functioning in the classroom, but on indicators of general functioning (internalizing problems; inattention/hyperactivity; emotional symptoms) which can probably be interpreted as previous aspects, not attributable simply to the arrangement of the class.

Another limitation is that not all the data collected were independent from one another. Indeed, the analyses conducted (*t*-tests) were based on the premise of non-independence of the data. This limitation could have been circumvented by introducing group affiliation as a covariate in the analyses. Furthermore, the gender-differentiated analysis used separate *t*-tests, but this distinction required first validating that significant interaction emerged *a priori*. This would have required conducting a MANOVA predicting mental health indicators and wellbeing and including gender and group as inter-subject variables. Were this interaction significant, separate *t*-tests would have been indicated. However, given the gender significance of the results, there is little doubt that this interaction was significant. The other limitation of our study concerns the limited literature on the topic. Indeed, our results could not be documented and supported by other studies that conducted gender-differentiated analyses. The final limitation of our study is that we did not consider students with special needs. A study should focus on the inclusion of these students in the context of flexible seating classroom.

Therefore, the above considerations need to be confirmed. Ultimately, this study had an exploratory intention. In addition, the protocol had to be modified because of COVID-19, a measurement time could not be completed. As we cannot redo the study, we can only add limits to the discussion and place the study in an exploratory context of research on a seed grant model in a new field.

### Prospects for Research

Our findings suggest the need for further studies on the topic. Indeed, the results of this study provide initial data on the influence of classroom seating arrangement on student wellbeing and mental health at school.

In view of the differences found between boys and girls, it would seem vital to make gender-differentiated analyses routine in scientific research related to human health or behavior (Tannenbaum et al., 2019). In the future, it would be relevant for studies conducting qualitative analyses to gather student and teacher perceptions to have a better understanding of school design-related factors influencing student mental health and wellbeing.

Also, it should be emphasized that the pedagogical methods within the classrooms affect a positive modification of the learning processes in children compared to the fixed seating classroom. In fact, in this sense, in a future study, pedagogy and learning processes should also be investigated in addition to wellbeing.

Finally, it would be very interesting in future studies in this field to favor a comparison between different cultures with an intercultural perspective. Thus, flexible classrooms could promote inclusive processes for children with special educational needs.

## Data Availability Statement

The raw data supporting the conclusions of this article will be made available by the authors, without undue reservation.

## Ethics Statement

The studies involving human participants were reviewed and approved by Institutional Human Research Ethics Committee (CIEREH) of the Université du Québec à Montréal (UQAM) and bears the following ethics certificate number: 3761_e_2019. Written informed consent to participate in this study was provided by the participants’ legal guardian/next of kin.

## Author Contributions

All authors listed have made a substantial, direct, and intellectual contribution to the work and approved it for publication.

## Funding

This study was funded by the Social Sciences and Humanities Research Council of Canada (SSHRC) under an Insight Development Grant (reference number: 430-2019-00875).

## Conflict of Interest

The authors declare that the research was conducted in the absence of any commercial or financial relationships that could be construed as a potential conflict of interest.

## Publisher’s Note

All claims expressed in this article are solely those of the authors and do not necessarily represent those of their affiliated organizations, or those of the publisher, the editors and the reviewers. Any product that may be evaluated in this article, or claim that may be made by its manufacturer, is not guaranteed or endorsed by the publisher.

## Footnotes

^1^“discours pédagogique actuel […] axé sur l’apprentissage, sur le fait de mettre l’élève au centre du débat, de l’aider à s’adapter, à être créatif, coopératif, réactif et autonome.” [Our translation].

^2^“ressentir une grande satisfaction générale à l’égard de sa vie.” [Our translation].

^3^“le bien-être devrait être compris comme un état de plaisir subjectif et de satisfaction à l’égard de la vie, mais aussi de réalisation de soi.” [Our translation].

^4^“l’étude scientifique des expériences positives, du bien-être et du fonctionnement optimal de l’individu.” [Our translation].

^5^“Je pense que de bonnes choses vont m’arriver dans ma vie”; “Je m’entends bien avec les gens”; “Je me suis sentie détendue.” [Our translation].

^6^“Je fais souvent des choses sans réfléchir”; “L’école ne m’intéresse pas”; “J’aime qui je suis” [Our translation].

^7^“Je m’entends bien avec les autres”; “Je suis nerveux”; “Je sais bien écouter.” [Our translation].

## Abbreviations

CIEREH: Institutional Human Research Committee (Comité institutionnel d’éthique de la recherche avec des êtres humains)
CSS: School Services Centre (Centre de services scolaire)
CSE: Higher Education Council (Conseil Supérieur de l’Éducation)
SD: Standard Deviation
IMSE: Socio-economic Background Index (Indices du milieu socio-économique)
M: Mean
